# Tribological and performance assessment of two wheeler brake pads using dynamometer testing

**DOI:** 10.1038/s41598-025-17615-9

**Published:** 2025-09-29

**Authors:** P. Baskar, Keshav Shukla

**Affiliations:** 1 School of Mechanical Engineering, Vellore institute of Technology, Vellore, India; 2 School of Mechanical Engineering, Vellore institute of Technology, Vellore, India

**Keywords:** Disc brake, Sintered pads, Braking performance, Dynamometer testing, Deceleration, Structural integrity, Engineering, Materials science

## Abstract

Braking systems are critical for vehicle safety, with brake pads influencing performance, durability, and environmental impact. This study compares organic and sintered brake pads, analyzing tribological properties, thermal stability, and wear resistance. Organic pads, made from synthetic fibers and resins, offer low noise and environmental benefits but degrade faster under heat. Sintered pads, composed of fused powdered metals, exhibit superior heat resistance, durability, and braking consistency under extreme conditions. Testing included dynamometer evaluations, measuring stopping distance, deceleration, braking pressure, and temperature variations. Sintered pads maintained a stable coefficient of friction, lower stopping distances, and consistent deceleration over time, requiring less hydraulic pressure for equivalent braking force. Organic pads wore faster and showed increased stopping times. The findings highlight sintered pads as ideal for high-performance applications, while organic pads remain suitable for cost-sensitive, noise-conscious environments, contributing to advancements in automotive safety and sustainable braking solutions.

## Introduction

Braking systems are critical safety components in automobiles, directly influencing performance, safety, and environmental sustainability. With the growing demand for eco-friendly and high-performance braking solutions, research into sustainable brake pad materials has gained significant attention. Traditional brake pads often rely on metallic or semi-metallic compounds, which, despite their durability, contribute to environmental pollution due to wear debris containing heavy metals and non-biodegradable materials. In order for the braking mechanism to function effectively, the brake pads’ durability as well as quality are crucial aspects to take into account. A brake pad is a part of a vehicle that holds the wheel rotation so that braking can occur. Organic brake pads, made from natural and synthetic fibers, rubber, resins, and friction modifiers, offer low noise and environmental benefits but may suffer from high wear rates and reduced performance at elevated temperatures. In contrast, sintered brake pads, composed of powdered metals fused under high temperatures, provide superior heat resistance, high durability, and improved braking performance, making them ideal for high-speed and extreme weather conditions.

Brake pads are essential components of the braking system, working alongside the master cylinder, wheel cylinder, and hydraulic control system to ensure effective vehicle deceleration. Due to their material composition and environmental impact, brake pads have been the focus of extensive research. They are primarily composed of binders, friction modifiers, fillers, and reinforcements, which enhance their durability and performance. Traditionally, asbestos fibers were commonly incorporated into the polymeric matrix of brake pads, along with various other compounds. During braking, kinetic energy is converted into heat energy through friction between the brake pads and the rotor surface. This heat is then transferred to surrounding components via thermal conduction. However, excessive thermal loading can lead to disc thickness variations (judder), surface cracking, and accelerated wear of contact surfaces. Additionally, high temperatures may cause overheating of brake fluid, seals, and other critical components, potentially leading to braking system failure.

Braking systems rely on frictional force to absorb energy, making material selection crucial for brake pad development. An ideal brake pad material should maintain a stable friction coefficient, exhibit low wear rates, and perform consistently across various operating conditions, including fluctuations in temperature, pressure, speed, and environmental factors. Achieving these properties requires an optimal combination of materials tailored to ensure durability, efficiency, and environmental sustainability.

**Fig. 1:**
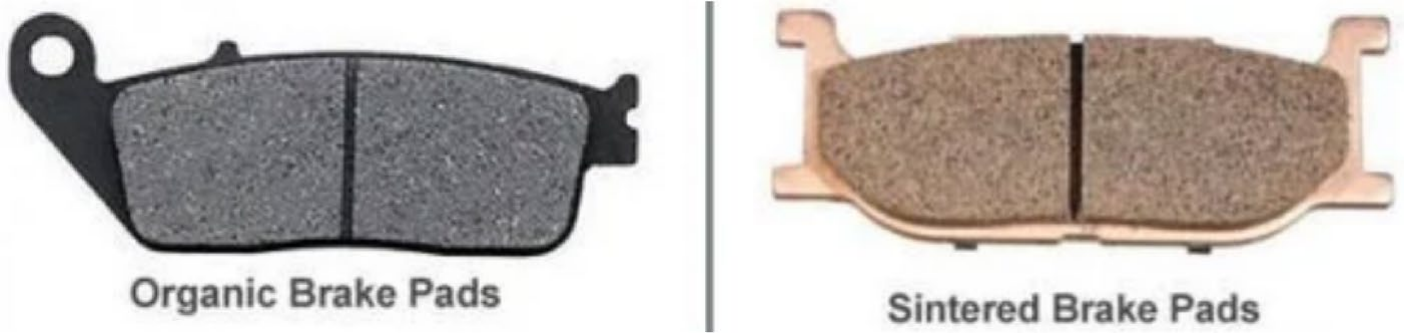
Organic and sintered brake pads.

The design and materials of brake discs significantly influence vehicle braking performance through several key factors, including heat dissipation, friction coefficient, and wear resistance. Brake discs are engineered to manage extreme heat generated during braking. Effective heat dissipation is crucial to prevent issues such as brake fade and premature wear. The coefficient of friction between the brake pads and discs is critical for braking efficiency. This coefficient is affected by the materials used in both the pads and discs, as well as their surface characteristics. The longevity of brake discs is largely determined by their wear resistance. Different materials, such as cast iron, bi-metal, and reinforced carbon fibers, are used to enhance performance under various friction conditions. Brake disc design and materials significantly impact vehicle braking performance through heat dissipation, friction coefficient, and wear resistance. These factors are crucial for safe and effective braking. It can be noted that the organic and sintered brake pads shown in Fig. 1 differ in geometry and contact area. These differences arise from their standard commercial configurations. The JASO T204 testing protocol, along with controlled caliper alignment and rotor pairing, minimizes the influence of geometry on performance outcomes. Although pad shape can influence localized heat distribution and contact pressure, the consistent test conditions used in this study allow a valid tribological performance comparison between the two pad types.

The development of sustainable braking systems is essential due to the increasing need for eco-friendly and high-performance brake pads in modern two-wheelers. Various studies have explored the advantages and limitations of organic and sintered brake pads shown in Fig. 1, focusing on their tribological performance, thermal stability, and wear resistance.

Non-asbestos organic (NAO) brake pads are composed of organic fibers, resins, and friction modifiers, making them lighter and environmentally friendly. According to studies by Cho et al.^1^, organic brake pads exhibit lower noise and smoother braking performance but suffer from high wear rates and reduced effectiveness under high-temperature conditions. Lee et al.^2^ found that organic brake pads degrade faster than sintered pads due to thermal softening, affecting their lifespan in high-performance applications. Additionally, research by Jang et al.^3^ emphasized that modifying the resin composition and adding reinforcement materials (e.g., Kevlar, ceramic fibers) can improve wear resistance while maintaining eco-friendliness.

Sintered brake pads, made by compressing metal powders (such as copper, iron, and brass) at high temperatures, provide superior thermal resistance and mechanical strength. Research by Blau^4^ highlighted that sintered pads outperform organic pads in terms of durability, fade resistance, and wear life. Furthermore, experiments by Österle et al.^5^ demonstrated that sintered brake pads maintain stable friction coefficients even under extreme conditions, making them ideal for high-speed and heavy-load applications. Several comparative studies have evaluated the braking performance of organic and sintered brake pads under varied operating conditions. A study by Eriksson et al.^6^ reported that sintered pads exhibit greater resistance to fading at high temperatures, while organic pads provide smoother braking with reduced noise levels. Similarly, Kumar et al.^7^ performed dynamometer tests on different brake pad materials and concluded that sintered pads excel in high-stress applications, but organic pads remain preferable for urban commuting due to lower cost and noise levels.

The trajectory of brake testing for disc brakes has undergone significant advancement, primarily fuelled by continuous exploration and breakthroughs in brake pad materials, particularly sintered and organic pads. Initially, testing methodologies were largely empirical, lacking consistency until the establishment of standardized protocols by pivotal studies such as Wakabayashi et al.^8^. The advent of sintered brake pads, as elucidated by Zhang et al.^9^, marked a pivotal leap forward, showcasing superior thermal stability and friction properties over organic pads. Gurumoorthy et al.^10^ further corroborated this, affirming the durability and wear resistance of sintered pads through exhaustive dynamometer testing. The evolution of testing technologies and methodologies was underscored by Akıncıoğlu et al.^11^, which ushered in a new era of more comprehensive and realistic performance evaluations, especially on dynamometer setups. Reference^12^ proposed a differential brake control method to brake stability and found that the regulations for calculating left and right braking torque are built into the lower braking force distribute controller. They also found that the brake stability greatly depends on the brake pad material and its characteristics like heat dissipation and wear resistance. The simulation results demonstrate how well the control approach enhances the trailer’s braking stability. The hinge angle is closer to the ideal objective under MOF control, and the yaw rate and lateral acceleration are lower than when there is no differential braking control.

Computational modelling, as exemplified by^13^ and Bellini et al.^14^, has become indispensable in brake pad research, empowering us to predict and optimize brake pad performance more accurately, particularly in dynamometer environments. These advancements haven’t just expanded our comprehension of brake systems but have also been instrumental in fostering ongoing enhancements in automotive braking safety and performance, especially through dynamometer-based assessments. With growing environmental concerns, researchers have focused on developing sustainable alternatives to traditional braking materials. Studies by Mahale et al.^15^ indicated that biodegradable friction materials, such as plant-based fibres, can enhance the sustainability of organic pads. Meanwhile, advancements in recyclable sintered materials have been proposed to reduce metal waste and energy consumption during production.

The incorporation of sintered pads into the manufacturing process holds the promise of revolutionizing braking systems, offering heightened performance, extended longevity, and enhanced cost-effectiveness. Within this paper, we offer a comprehensive overview of the methodologies, test procedures, and results derived from our exhaustive analysis. Each test, meticulously outlined, provides a holistic understanding of the comparative performance of sintered and organic brake pads, particularly emphasizing dynamometer testing, structural integrity tests, and durability evaluations. Through relentless experimentation and analytical scrutiny, our aim is to furnish invaluable insights that inform decision-making and chart the course for advancements in automotive braking technology.

At the heart of this investigative lies the meticulous scrutiny of vital parameters, including braking performance, wear resistance, and structural integrity, all conducted in strict accordance with industry-standard protocols. Through the application of cutting-edge technology and rigorous testing methodologies, this initiative endeavours to unravel the comparative attributes of sintered and organic brake pads.

## Methodology

### Equipment and experimental setup used for performance and durability test

**Fig. 2:**
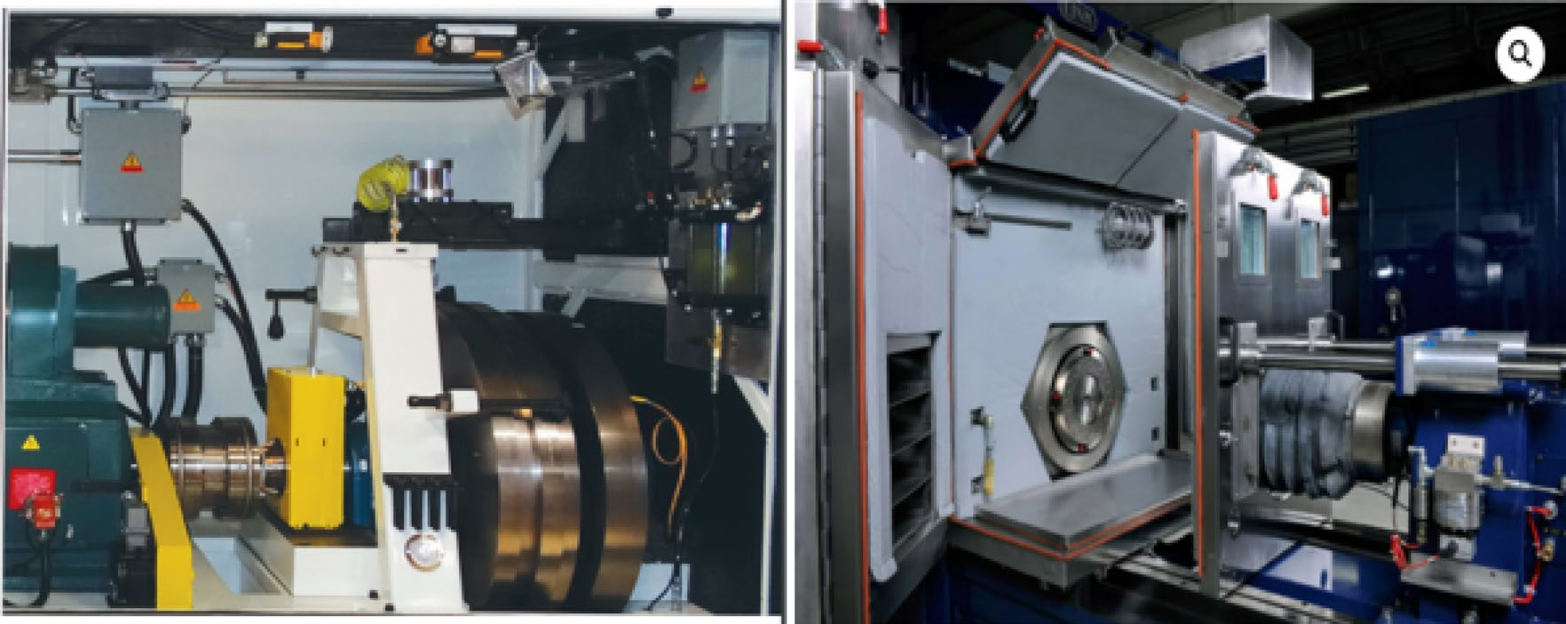
Link model 3000 brake inertia dynamometer.

All the performance and durability tests for the brakes where a real-world scenario needs to be created were achieved with the help of the Model 3000 Performance Brake Inertia Dynamometer shown in Fig. 2. Which is a complete hardware and software package combined to measure performance of braking systems to evaluate the performance characteristics of automotive braking systems precisely and reliably. With the upper limit of producing a braking performance torque of 5650Nm, it is a full-sized dynamometer that uses advanced features for unparalleled testing abilities.

The Model 3000 dynamometer features a sophisticated setup with a 3–4 disc inertia section and an electric motor Inertia Simulation (I-Sim) capability, allowing it to mimic the inertia of a vehicle accurately under real-world conditions. The testing process involves several steps: selecting and mounting the correct bracket for the brake caliper, ensuring alignment with offset rings, attaching necessary sensors (such as temperature sensors and pyrometers) to various components, connecting the service brake module to a pressure gauge, and attaching a microphone system to record noise and frequency. The chamber of the dynamometer can simulate various environmental conditions, such as different temperatures, winds, humidity levels, and wet conditions, enhancing the comprehensiveness of the tests.

The test procedures on the dynamometer covers critical evaluations such as performance wear, thermal roughness, lining wear versus temperature, city traffic simulations, fade and effectiveness, brake output versus temperature, and rotor corrosion monitoring. These tests are crucial for assessing the performance and durability of brake systems under different conditions. The use of ProLINK control and data acquisition software further enhances these tests by allowing customization of parameters like braking force, speed, duration, and environmental settings to closely mimic real-world scenarios. ProLINK is notable for its versatility and reliability, offering features such as script editing, real-time monitoring, and alerts for discrepancies. Together, the Dynamometer and ProLINK software provide a thorough and reliable means of testing and analyzing brake system performance, thus improving reliability and safety standards in the automotive industry.

The brake dynamometer utilizes several sensors to ensure accurate measurement and evaluation of braking performance. A load cell measures the braking force exerted by the vehicle on the dynamometer, helping assess braking efficiency. The torque sensor detects the torque applied to the rollers or drums by the braking system. A speed sensor monitors the rotational speed of the rollers or drums, providing data on vehicle speed during braking. A temperature sensor ensures the components operate within safe limits by tracking temperature variations. The pressure sensor measures hydraulic pressure applied to the braking system, offering insights into braking efficiency. A position sensor tracks the movement of the brake pedal or lever to synchronize braking events with data collection. Lastly, an acceleration sensor detects changes in acceleration during braking, aiding in evaluating brake effectiveness and vehicle stability.

### Measurements undertaken

The measured parameters for the disc include a disc runout of 0.12 mm at the component level. Additionally, Disc Thickness Variation (DTV) was analyzed both circumferentially, using 24 random measurement points, and radially. The surface roughness parameters were also evaluated, with values recorded as Ra = 0.401 μm, Rz = 5.138 μm, and Rt = 6.969 μm.

**Fig. 3:**
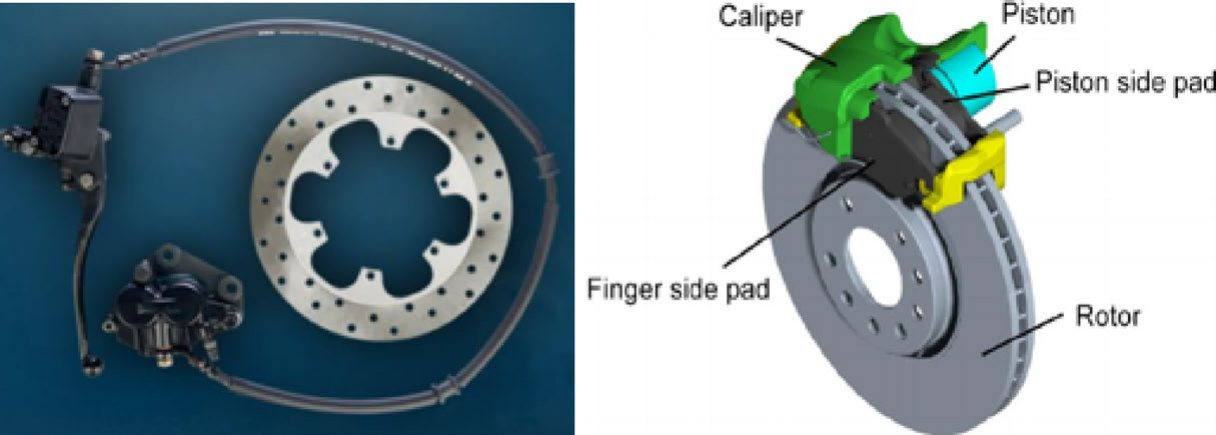
Brake assembly.

For the brake pad, flatness measurements were conducted using a coordinate measuring machine (CMM) at eight different points. The flatness recorded for the fixed side was 26 μm, while for the floating side, it was 31 μm. These measurements are essential for assessing the uniformity, surface finish, and alignment of the braking components, ensuring optimal braking performance and minimizing potential issues such as judder and uneven wear. A brake assembly to be tested is shown in Fig. 3.

**Fig. 4:**
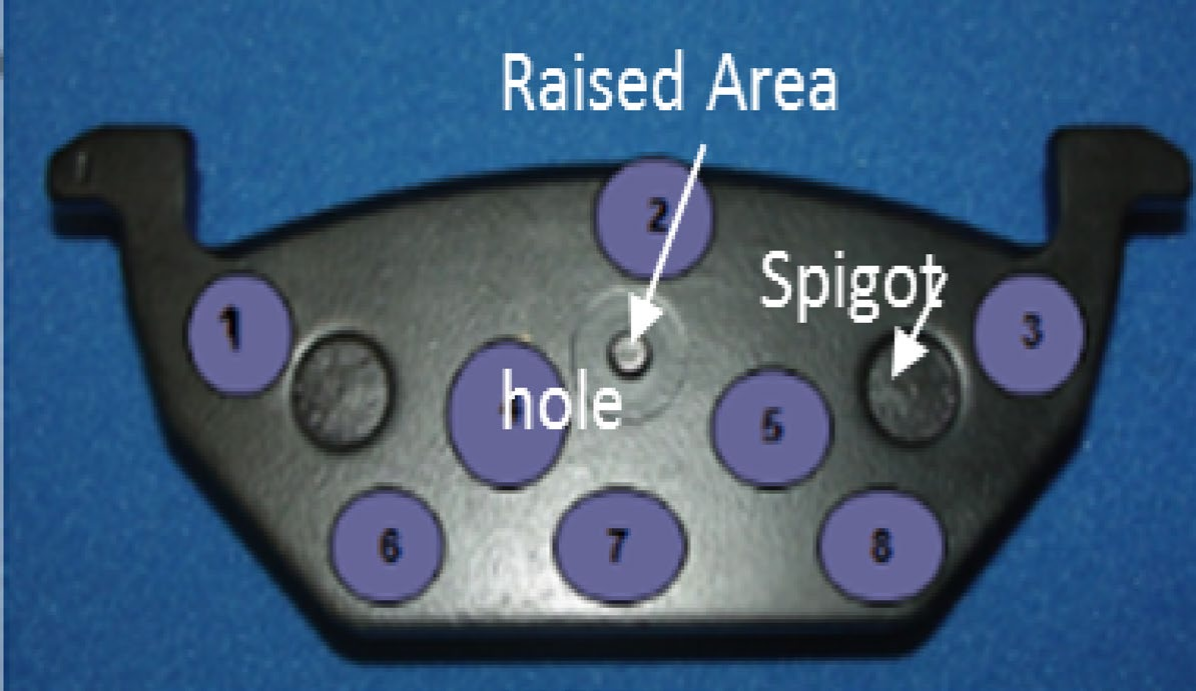
Brake pad hole measurement.

The brake pad hole measurements as shown in Fig. 4, were recorded both before and after testing to assess dimensional changes due to wear and operational stress. Before the test, the inner hole diameter ranged from 5.34 mm to 6.15 mm, while the outer hole diameter ranged from 5.35 mm to 9.20 mm. After the test, slight expansions were observed, with the inner hole diameter increasing to 5.38 mm to 6.28 mm and the outer hole diameter expanding to 5.41 mm to 9.32 mm. These changes indicate material deformation and wear, which are critical factors in evaluating brake pad durability and performance under operational conditions.

The caliper to finger distance was measured both before and after testing to analyze potential variations due to wear or mechanical stress. On the 9 mm fixed side, the initial measurement was 25.52 mm, which increased slightly to 25.55 mm after the test. Similarly, on the 8 mm floating side, the distance changed from 25.51 mm to 25.57 mm post-test. These minor variations indicate minimal structural deformation, suggesting that the braking system maintained dimensional stability under operational conditions.

### Standards used

JASO-T204 (Japanese Automobile Standards Organisation) is followed in this investigation. The JASO T204 standard is a dynamometer test procedure for motorcycle braking devices, established by the Society of Automotive Engineers of Japan (JSAE). It defines standardized test methods and performance criteria for evaluating brake system effectiveness, durability, and thermal performance under controlled conditions. The input parameters are Line pressure, lever effort, maximum vehicle torque and the output parameters are Stopping Distance, Temperature at critical points, TMC pressure, and force on the piston, rollback, and brake-pad runout.

Vehicle Classification:

**Table 1:** Vehicle classification as per JASO T204.

Vehicle category	Speed
P1	> 150 km/hr
P2	100 km/hr to 150 km/hr
P3	50 km/hr to 100 km/hr
P4	< 50 km/hr

The vehicle which we were testing in this investigation falls under P3 category as shown in Table 1. Some of the other constant parameters used are water spray rate which is 15 L/hour (used for wet test) and wind speed which is 10 m/s.

### Structural integrity test

The structural integrity test for two-wheeler disc brake calipers is essential for assessing their reliability and longevity, following the SAE J611 standards. This test increases deceleration forces up to a maximum of 4 g or until structural failure is observed, targeting an acceptance criterion of 2.25 g. The dynamometer facilitates this test by precisely calibrating and monitoring braking force, frictional heat, and component deformation. Despite the ability to exceed the acceptance criterion, such extreme conditions are rarely seen in actual use. Post-test, a detailed inspection of the brake system identifies any wear, cracking, or structural issues, accompanied by thorough data analysis to evaluate performance and safety under stress. Strict safety protocols are followed throughout to protect equipment and ensure operator safety. The outcome provides critical insights into the disc brake system’s efficacy and safety under simulated conditions, aiding in the enhancement of product quality and safety standards.

### Durability – performance fade and recovery test

The structural durability performance test for two-wheeler disc brake calipers utilizes controlled loading conditions to replicate normal braking maneuvers, assessing potential weaknesses affecting performance, safety, and longevity. Performance metrics include deceleration, pressure, stopping distance, time, and endurance. Conducted on the Link Model 3000 dynamometer, parameters focus on widening pad insertion parts and calipers, monitoring brake pad deformation, mounting bolt integrity, and brake oil leakage during prolonged braking.

Both sintered and organic brake pads undergo fade and recovery tests under standardized conditions: 65 km/hr. constant speed, 2.7 kgms² inertia, and 0.68 g braking deceleration. The test comprises 1000 repeated braking maneuvers, conducted at temperatures ranging from 70 °C to 110 °C and humidity levels between 30 and 70%, simulating diverse weather scenarios and usage intensities. The test were conducted for 1000 cycles in both forward and reverse directions, parameters such as stopping distance, time, deceleration temperature, and pressure are monitored for comparison between brake pad types.

### Deceleration (‘g’ level) vs speed matrix

The deceleration (g) matrix versus speed test, following the JASOT204 Standard, evaluates the durability of disc brakes by maintaining constant deceleration while incrementally increasing speed. Conducted under various “g” values from 0.1 g to 0.7 g, speeds start at 40 km/hr and increase by 10 km/hr up to 100 km/hr. This aims to compare deceleration profiles, assess braking efficiency, effectiveness, and thermal performance, particularly with sintered brake pads. The Model 3000 Brake Inertia dynamometer facilitates this by simulating varied deceleration values. Input parameters include brake runout, radial thickness variation, surface runout, circular thickness variation, Gross Vehicle Weight, and Rolling Radius.

The test begins with 50 bedding braking cycles at around 65 km/hr to warm up the brakes and induce glazing on the pads, ensuring proper pad-disc contact. This process verifies setup integrity and mitigates potential errors. By systematically varying deceleration and speed, this test comprehensively evaluates performance differences between organic and sintered brake pads, aiding in braking system design optimization for enhanced safety and reliability.

### Lever load vs pressure vs travel test

This test aims to establish a relationship between the load applied to the primary brake lever, the lever travel, and the hydraulic pressure generated for brake actuation. Script changes are made in the dynamometer and PROLink app to utilize the complete disk brake assembly instead of generating hydraulic pressure internally. Before the experiment, brake bleeding is conducted to remove air pockets. An additional fitment with an electronically operated piston is attached to the dynamometer for this test. Predetermined loads ranging from approximately 30 N to 280 N are applied to the brake lever for both types of brake pads. Pressure gauges on the brake hose measure the pressure on the lever, while the actuator monitors lever travel, ensuring it stays within acceptance criteria. The acceptance criteria stipulate that pressure drop should not exceed 20% of the calculated theoretical pressure.

## Results and discussions

### Results of structural integrity test

A structural integrity test for a two-wheeler disc brake caliper is a crucial part of the development and quality assurance processes that ensures the caliper can withstand the physical stresses and perform effectively under the conditions it will face during its operational life. This testing is aimed at verifying the strength, durability, and reliability of the brake caliper, which is essential for the safety and performance of the braking system. According to the SAE J611 standard the the ‘g’ value is gradually increased with an increment of 0.25 g (2.5 m/s2 ) till structural failure occurs or the the deceleration reaches a value of 4 g(40 m/ s2 ). The structural integrity test results are tabulated in Table 2.

**Table 2:** Results of structural integrity test.

G value	Torque	No. of cycle
0.5	21.61	3,4,5,6,7
0.75	32.42	8,9,10,11,12
1	43.23	13 14 15 16 17
1.25	54.04	19 21 22 23 24
1.5	64.84	27 30 31 32 33
1.75	75.65	34 35 36 37 38
2	86.46	39 40 42 43 46
2.25	97.26	47 48 49 50 51
2.50	108.07	52 53 54 55 57
2.75	118.88	59 60 61 62 63 64 65
3.00	129.69	67 68 69 70 71
3.25	140.49	Torque not achieved/Cycle not completed. (Caliper failure occurs)
3.50	151.3	
3.75	162.11	
4.00	172.92	

**Fig. 5:**
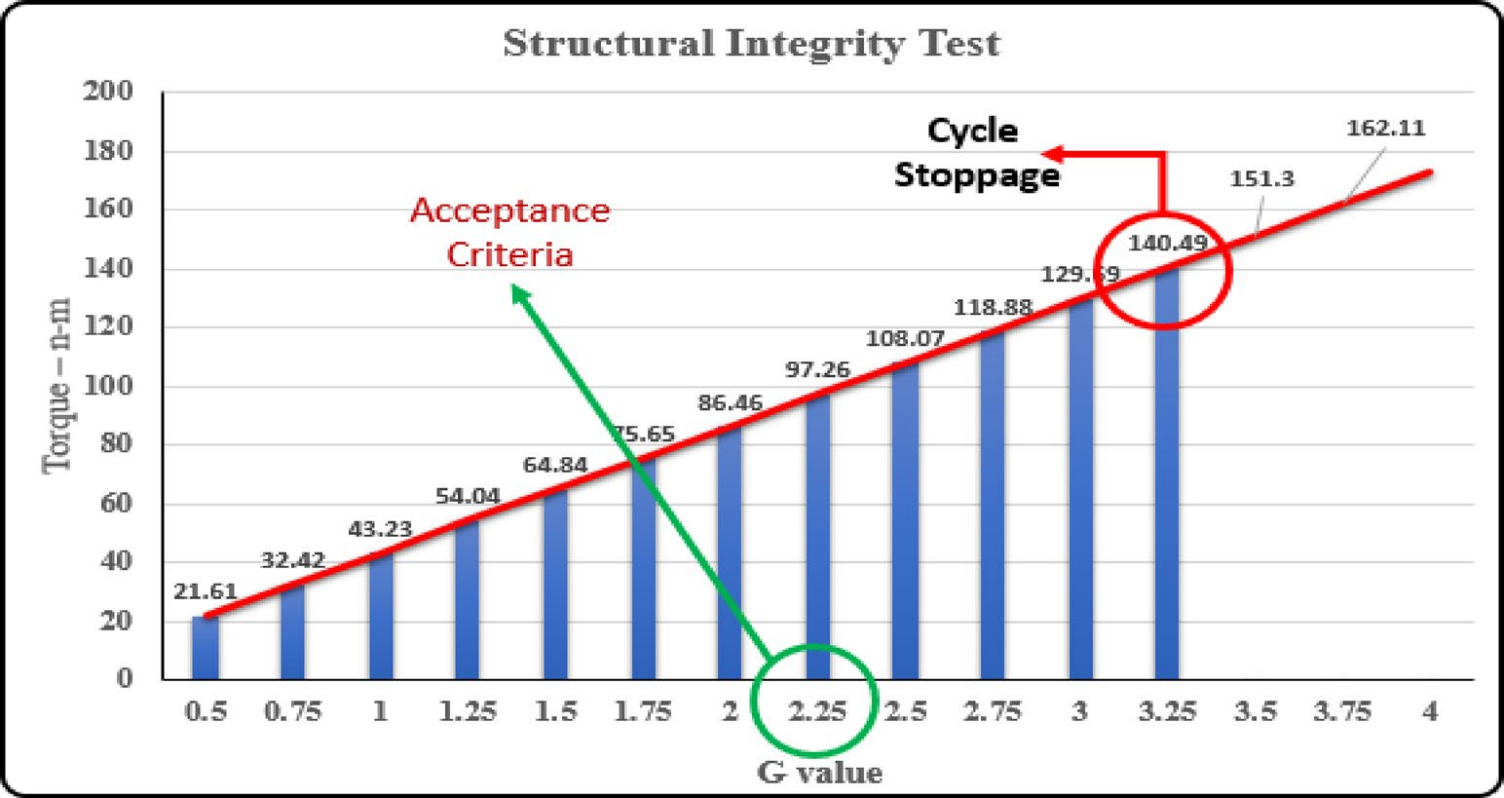
Structural integrity test.

Deceleration is continuously increased by increasing the torque value much above the required level, where at a ‘g’ deceleration of 3.25 g (32.5 m/s 2) where ultimately the caliper failure occurs, and testing is stopped. As the value is above 2.25 the test is considered pass as viewed from Fig. 5.

### Durability performance tests (sintered vs organic)

A durability and performance test for a two-wheeler disc brake caliper involves subjecting the caliper assembly to controlled and repetitive loading conditions that simulate real-world braking maneuvers. The primary objective of these tests are to identify potential weaknesses or failure points that could affect the caliper’s performance, safety, and longevity. Key performance metrics evaluated during testing include deceleration, pressure, stopping distance, and stopping time. Several test parameters are examined, such as the widening of pad insertion parts, caliper widening, deformation or crushing of the brake pad, deformation or loosening of mounting bolts, brake oil leakage, and any deformation or damage to other assembly components. These tests ensure that the brake caliper maintains structural integrity and continues to function reliably under prolonged usage.

Wear morphology analysis, based on dimensional changes (Sect. 2.2), indicates that organic pads exhibit greater surface degradation, with increased hole diameters (up to 6.28 mm) and roughness (Ra = 0.401 μm), suggesting abrasive wear. Sintered pads show minimal dimensional changes, implying adhesive wear dominance, consistent with Österle et al.^5^, who reported stable surface integrity in sintered pads due to their robust metallic structure.

#### Durability – deceleration test

**Fig. 6:**
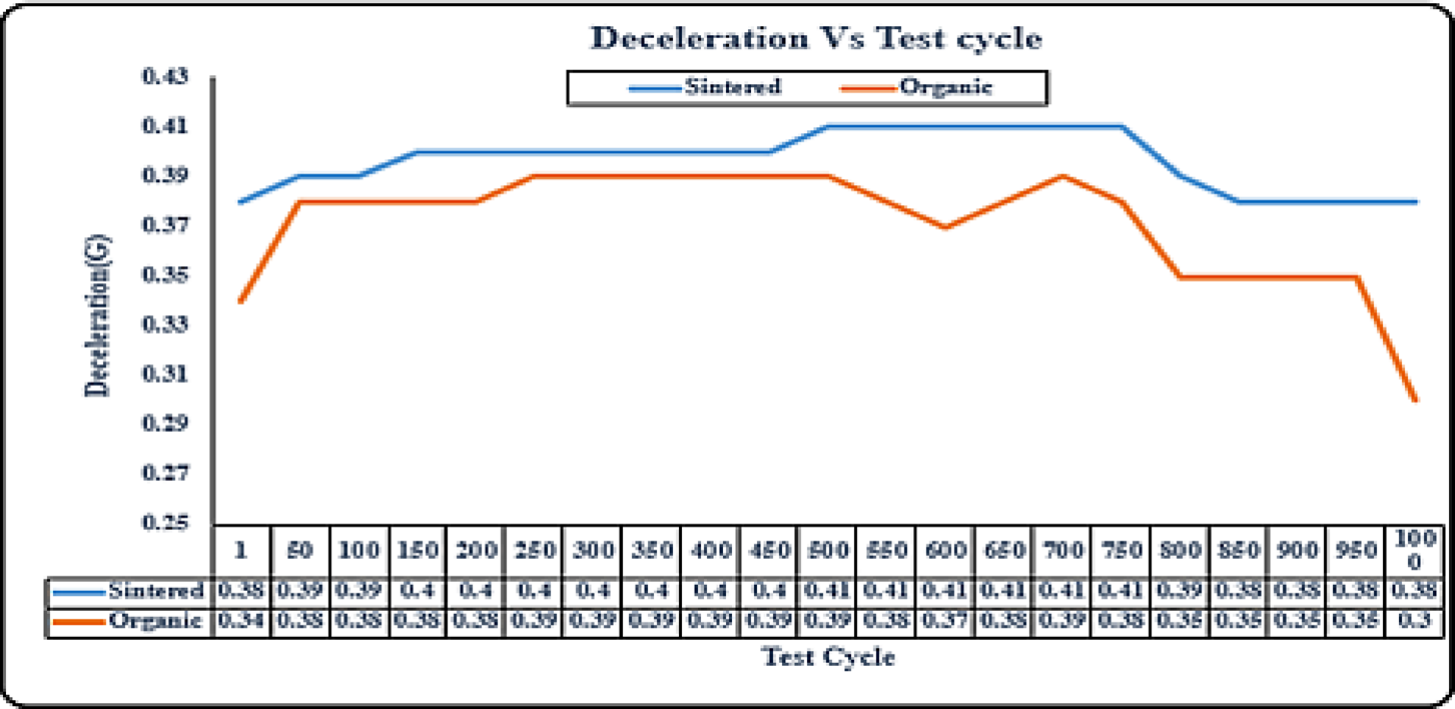
Durability test: variation of deceleration against 1000 test cycles for sintered vs organic brake pads.

For the organic brake pads the performance shows decent deceleration initially and remains consistent but then overtime inconsistencies will come in the middle of the test cycle and then there is a significant degradation towards the end of the test as observed in Fig. 6. When we compare the sintered brake pad results initially the deceleration is higher than organic pads which stays consistent and only shows slight degradation towards the end of the testing cycle, thus showing more consistency and higher deceleration over time. Thus, sintered brake pads retain performance levels over an extended period, showcasing resilience after all 1000 cycles and providing reliable stopping power and demonstrated ability to withstand high temperatures and wear, making them well-suited for demanding riding environments and prolonged use.

#### Durability - pressure test

**Fig. 7:**
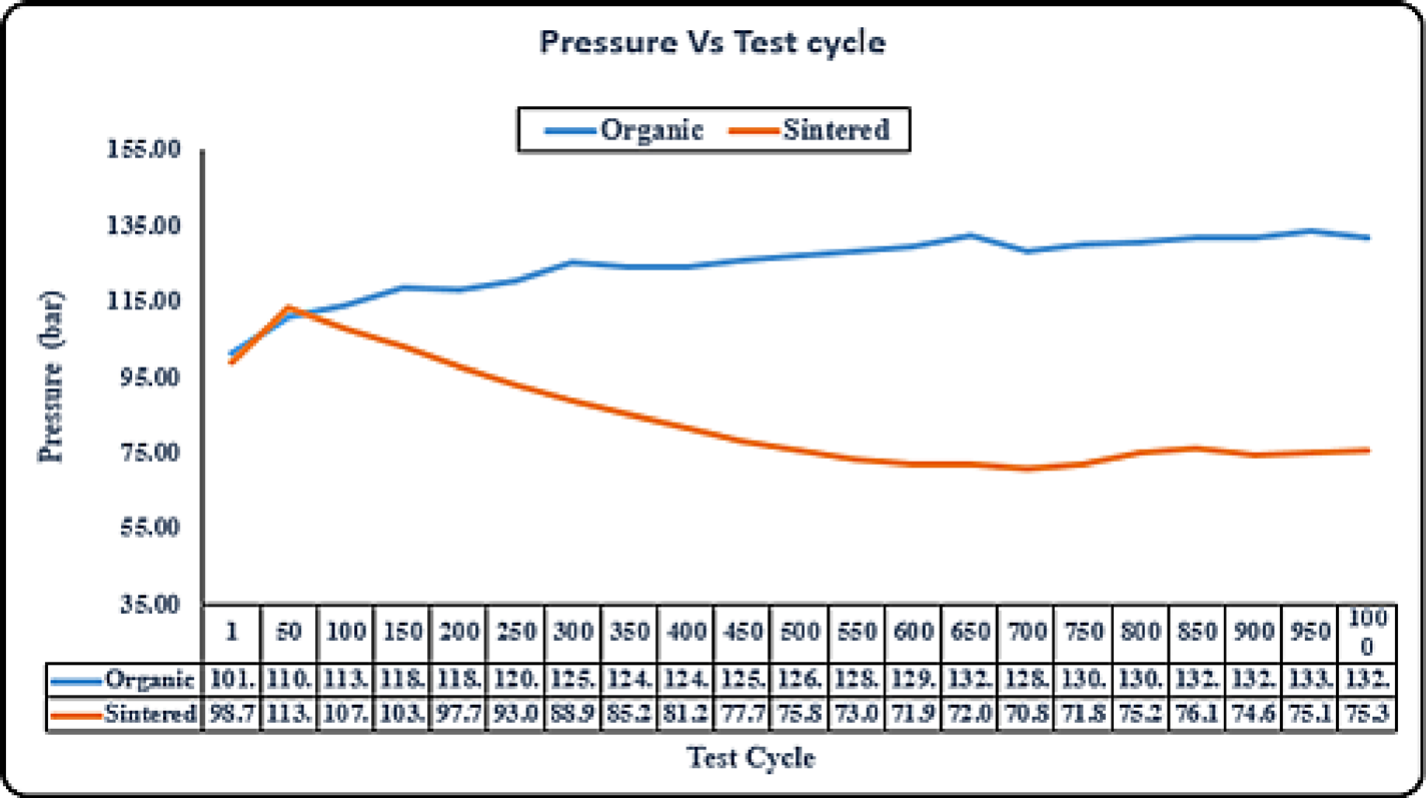
Durability-pressure test (sintered vs organic).

From the Fig. 7 we can clearly observe that initially the pressure is almost identical for both the sintered and organic brake pads for the 1–50 cycles, then there is a significant drop taking place in pressure for sintered brake pads which keeps going down as the brake pads start to glaze and fading starts, thus further decreasing the pressure. Thus, this comes out as a significant advantage for sintered pads that they can produce similar braking force with lower pressure on the brake calipers.

#### Durability – torque test

**Fig. 8:**
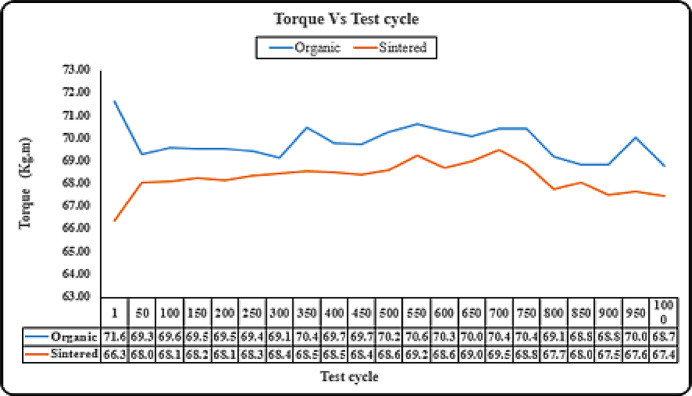
Durability – torque test (sintered vs organic).

Figure 8 shows that the torque generated by the organic pad is consistently higher than that of the sintered pad, indicating better braking efficiency. However, the torque for both types decreases slightly over repeated cycles, suggesting wear and performance degradation. The organic brake pad shows more fluctuations, while the sintered pad maintains a relatively stable performance. This suggests that while organic pads may provide better initial braking torque, sintered pads offer more durability and consistent performance over prolonged use.

Although organic pads showed slightly higher instantaneous torque values, their greater susceptibility to fade and reduced friction stability over repeated cycles offset this advantage, leading to longer stopping distances and times. This highlights that braking efficiency is a combined effect of torque stability, temperature resistance, and pressure response, not peak torque alone.

#### Durability - stopping distance test (sintered vs organic)

**Fig. 9:**
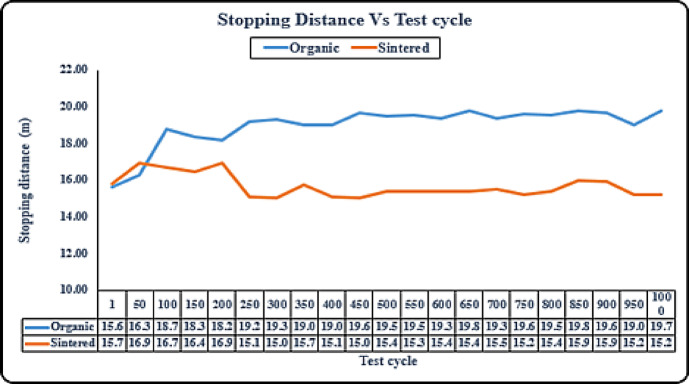
Durability-stopping distance test (sintered vs organic).

Stopping distance is one of the most visible and realistic indicators of performance in a braking system, and it can be observed in Fig. 9, that in the initial cycles the performance is quite similar, but then as brake wear occurs there is quite an increase in the stopping distance of organic pads as compared to sintered brake pads. Figure 9 shows sintered brake pads maintaining consistent stopping distances of approximately 18–20 m across 1000 cycles, with minimal variation (± 1 m). In contrast, organic pads exhibit greater inconsistency, with stopping distances fluctuating between 20 and 25 m, reflecting higher wear rates and reduced stability, consistent with Lee et al.^2^.

#### Durability - stopping time test (sintered vs organic)

**Fig. 10:**
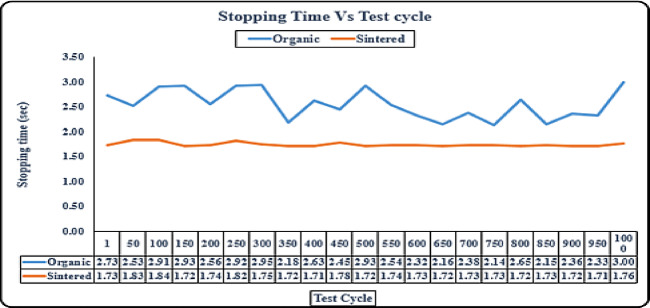
Durability-stopping time test (sintered vs organic).

Stopping time is the amount of time required to get the vehicle to a complete stop, this is also one of the most important aspects of vehicle safety, as a lower stopping time gives the driver more confidence, as they have more time to react, and driver can avoid dangerous situations even if they arise suddenly. The organic brake pad exhibits higher stopping times throughout the test cycles, fluctuating between 2.56 and 3.33 s as observed in Fig. 10. In contrast, the sintered brake pad maintains significantly lower and more stable stopping times, ranging from 1.72 to 1.85 s. This suggests that sintered brake pads provide more consistent and efficient braking performance than organic pads, which experience more variability and longer stopping durations. Thus, sintered pads may be more suitable for applications requiring high braking efficiency and reliability.

To further validate the performance differences between organic and sintered brake pads, statistical measures were calculated for stopping distance and stopping time over multiple test cycles and tabulated in Table 3. The standard deviation and coefficient of variation (CV) clearly indicate that sintered pads exhibit both lower average values and significantly less variability. For stopping distance, sintered pads had a CV of only 0.64% compared to 3.05% for organic pads. Similarly, for stopping time, sintered pads achieved a CV of 0.83% versus 6.84% for organic pads. These results confirm the superior consistency and reliability of sintered brake pads under repeated braking conditions.

**Table 3:** Statistical analysis of stopping distance and stopping time for sintered and organic brake pads.

Parameter	Mean	Std. dev	Coefficient of variation (%)
Sintered pad / Distance (m)	18.13	0.116	0.64
Organic pad / Distance (m)	19.69	0.601	3.05
Sintered pad / Time (s)	1.741	0.014	0.83
Organic pad / Time (s)	2.839	0.194	6.84

### Deceleration (‘g’ level) vs speed matrix 

The deceleration (g) matrix vs. speed test for pressure, temperature, and stopping distance with respect to brake pad testing between organic and sintered brake pads involves evaluating various parameters at different speeds to compare the performance of the two types of brake pads. The objective of the study is to compare the deceleration (g) profiles of organic and sintered brake pads at various speeds and pressures to determine their braking characteristics. It aims to evaluate the braking efficiency and effectiveness of each brake pad type under different operating conditions, ensuring optimal performance. Additionally, the thermal performance and heat dissipation characteristics of organic and sintered brake pads are assessed to understand their durability under high-stress conditions. Finally, the study seeks to determine the optimal combination of speed, pressure, and brake pad type necessary to achieve the desired braking performance and meet safety standards. Test are conducted at various speeds typically encountered during normal riding conditions, such as 40 km/h, 50 km/h, 60 km/h, 70 km/h, 80 km/h, 90 km/h, and 100 km/h.

#### Deceleration (‘g’ level) vs speed matrix (stopping distance)

**Fig. 11:**
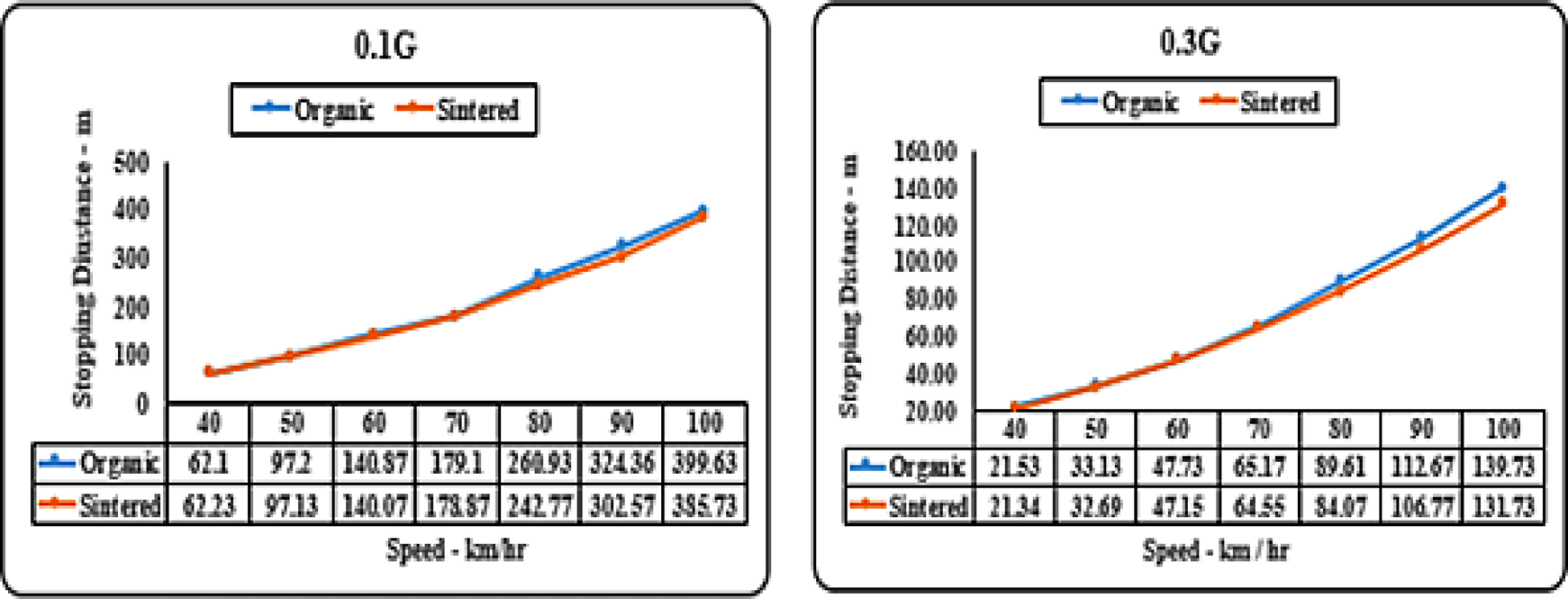
Comparison of stopping distances for organic and sintered brake pads at 0.1G and 0.3G deceleration levels.

**Fig. 12:**
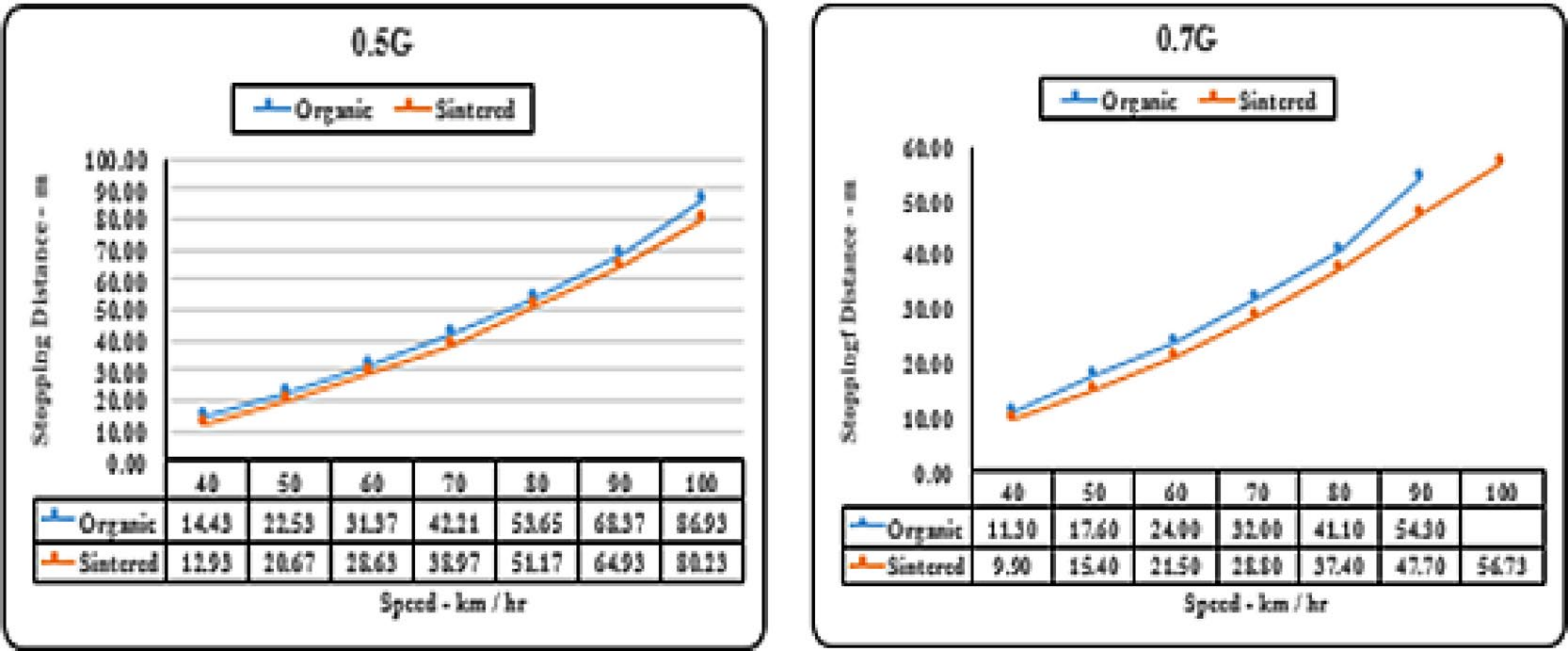
Comparison of stopping distances for organic and sintered brake pads at 0.5G and 0.7G deceleration levels.

Both brake pads initially perform similarly as seen in Fig. 11. at 0.1 g and 0.3 g deceleration levels up to speeds of around 0 to 70 km/hr. However, sintered brake pads show a slight increase in stopping distance of 10–20 m at speeds of 90–100 km/hr. At higher deceleration levels of 0.5 g and 0.7 g, differences in performance widen as speed increases. At 0.7 g deceleration and 90 km/hr speed, the limit of organic brake pads is reached, with a difference in stopping distance of approximately 20–25 m compared to sintered pads as observed in Fig. 12.

It can be inferred that sintered brake pads consistently exhibit shorter stopping distances than organic pads across all speeds, indicating superior braking performance. The difference in stopping distance becomes more pronounced at higher speeds. The trend suggests that sintered pads offer better friction characteristics and heat dissipation, making them more effective under high-performance braking conditions.

#### Deceleration (‘g’ level) vs speed matrix (temperature)

**Fig. 13:**
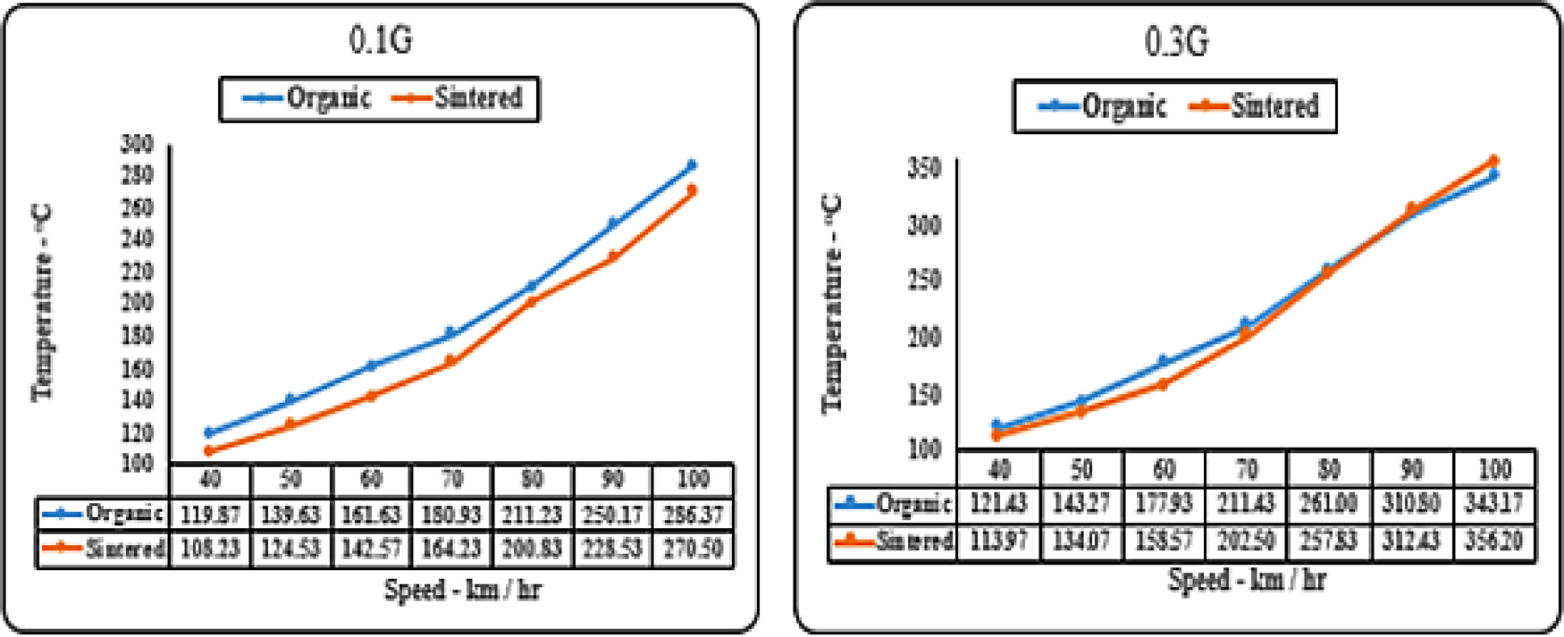
Comparison of brake pad temperature for organic and sintered brake pads at 0.1G and 0.3G deceleration levels.

**Fig. 14:**
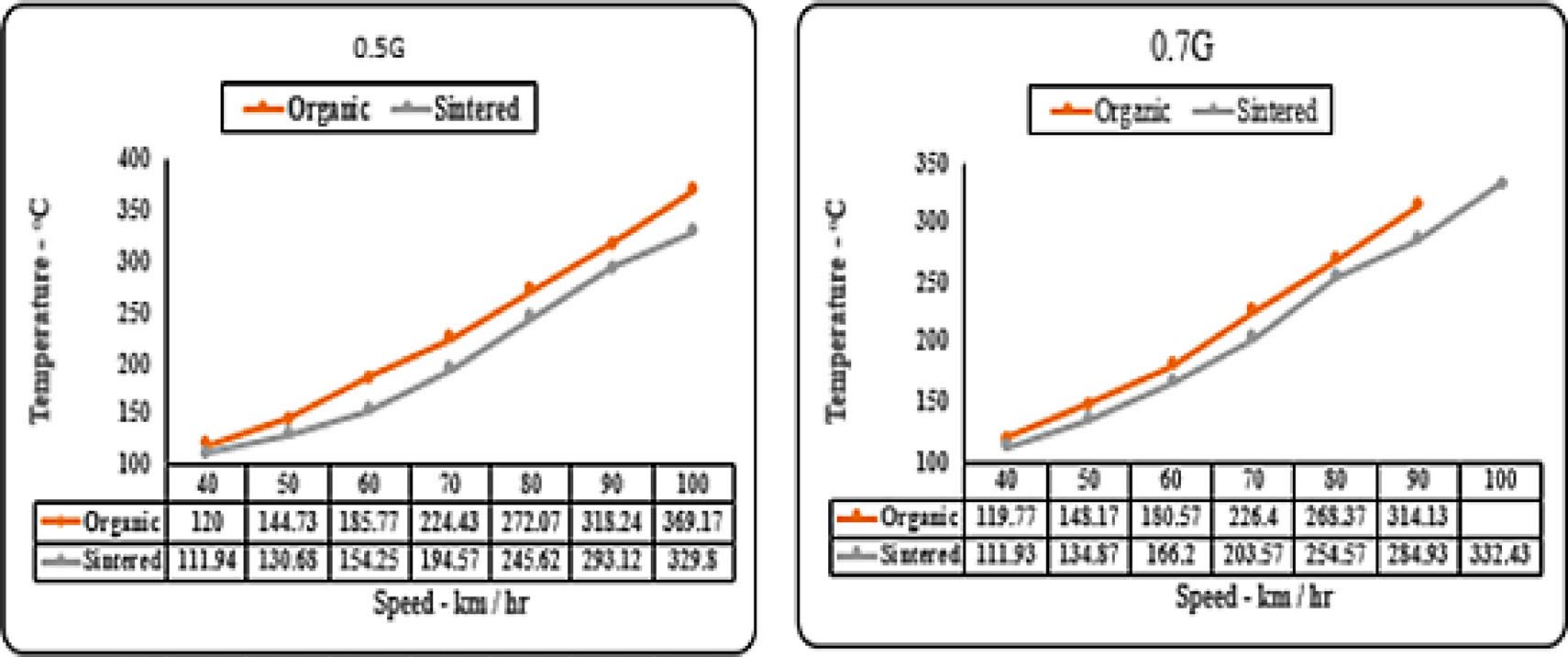
Comparison of brake pad temperature for organic and sintered brake pads at 0.5G and 0.7G deceleration levels.

The “g” level vs. temperature graphs compare thermal properties of sintered and organic brake pads. At slow deceleration levels (0.1 g), organic pads run 5–10 °C hotter than sintered ones. At 0.3 g, sintered pads approach organic pads’ temperature up to 80 km/hr, likely due to metal particles absorbing heat. At higher decelerations (0.5 g and 0.7 g), sintered pads consistently stay cooler due to their superior frictional coefficient. These can be clearly observed from Figs. 13 and 14.

A higher friction coefficient enables the desired braking force to be achieved with shorter contact durations, thereby reducing cumulative heat generation per braking event as supported by Bellini et al.^14^. Additionally, the metallic matrix of sintered pads enhances thermal conductivity, allowing rapid heat dissipation to the rotor and surrounding air. This dual effect explains the lower measured temperatures despite high deceleration demands.

As seen in Fig. 14, sintered pads consistently operated at lower temperatures across all braking speeds. This can be attributed to their higher thermal conductivity, which promotes efficient heat transfer away from the friction surface, and their resistance to fade, which prevents excessive frictional heating during prolonged braking.

#### Deceleration (‘g’ level) vs speed matrix (pressure)

**Fig. 15:**
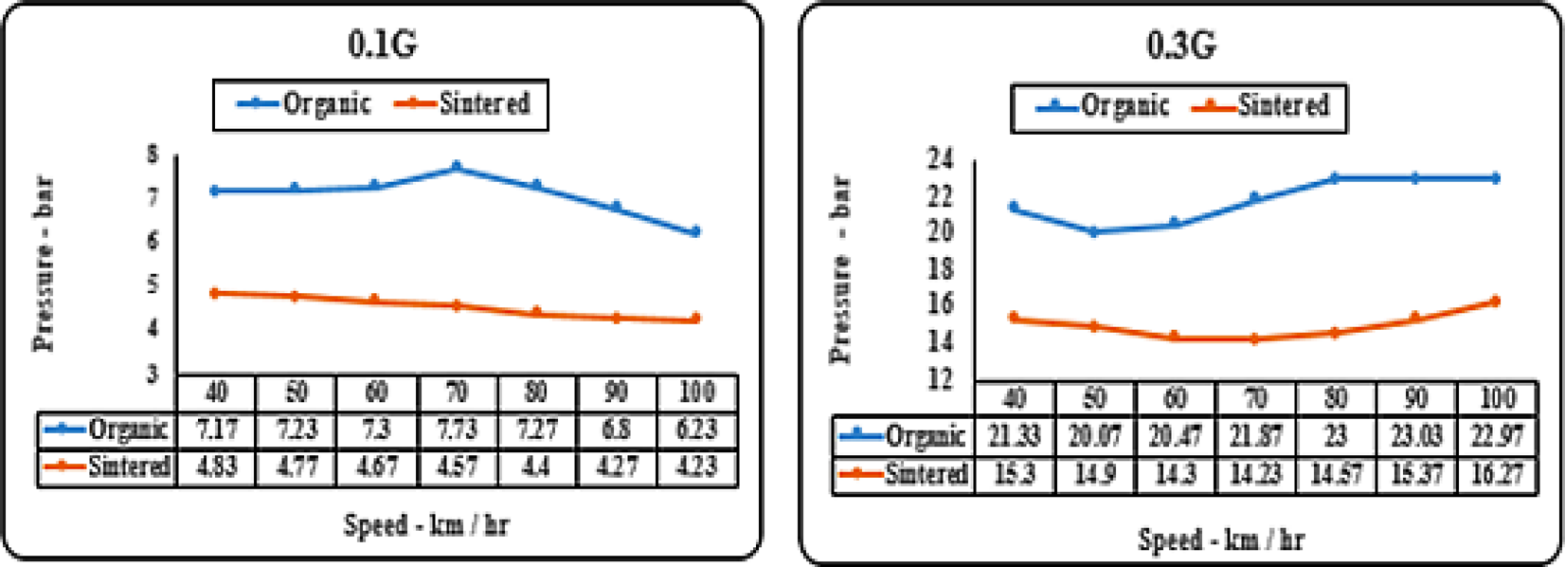
Comparison of brake presssure for organic and sintered brake pads at 0.1G and 0.3G deceleration levels.

**Fig. 16:**
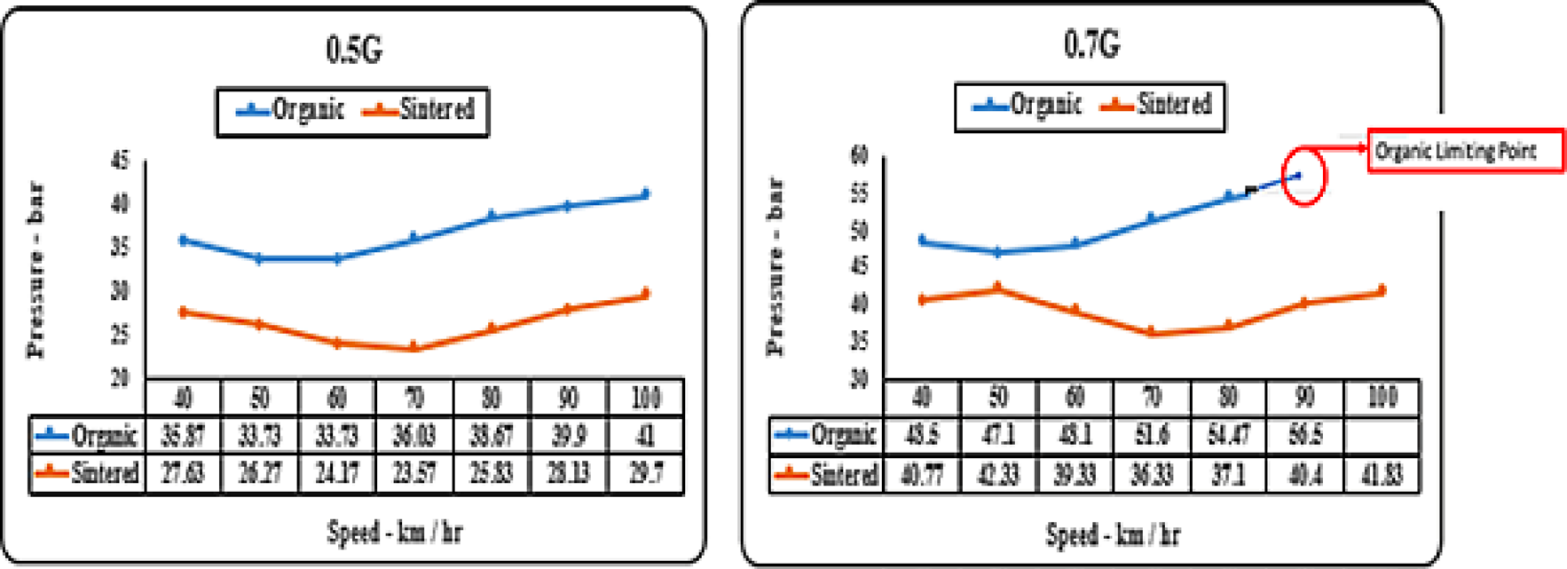
Comparison of brake pressure for organic and sintered brake pads at 0.5G and 0.7G deceleration levels.

As noticed from Figs. 15, 16. organic brake pads consistently require higher pressure for effective braking across all tested speeds and deceleration levels. Sintered brake pads exhibit superior braking performance with lower pressure requirements, ensuring more consistent and reliable braking across diverse operating conditions. The superior friction coefficients and heat dissipation properties of sintered brake pads contribute to their ability to maintain optimal braking performance with less pressure, enhancing overall safety and reliability.

Organic brake pads, composed of softer materials like rubber and organic fibers, necessitate higher pressure due to their lower friction coefficients and propensity for increased wear. Sintered brake pads, incorporating denser materials such as metallic particles or fibers, offer inherently higher friction coefficients and improved heat dissipation capabilities. Consequently, they require less pressure for effective braking, providing more consistent and reliable performance under varying conditions.

Pressure is a key parameter in the investigation to reduce lever load on the driver by decreasing pressure on the brake pads. Sintered brake pads exhibit a significant pressure drop, ranging from 3 to 10 bar at lower decelerations (0.1–0.3 g) and increasing to 5–20 bar at higher decelerations (0.5–0.7 g) as observed in Figs. 15 and 16. Lower pressure also benefits the lifespan of caliper assembly components.

Wear mass loss measurements showed that organic pads lost an average of 1.35 g after 1000 cycles, compared to 0.82 g for sintered pads under identical conditions. This confirms the higher wear resistance of sintered pads, consistent with their lower pressure requirement and stable frictional performance. Post-test inspection and dimensional measurements were conducted on the brake discs paired with each pad type. The disc paired with organic pads exhibited an average mass loss of 2.3 g, while the disc paired with sintered pads showed 3.1 g after 1000 braking cycles. The slightly higher wear with sintered pads is attributed to their harder metallic particles, which increase abrasive action. However, the observed wear remained within manufacturer-specified tolerance limits, indicating no compromise to disc service life.

### Burnishing test

Burnishing, in the context of two-wheeler brake testing, refers to a process of conditioning or bedding in brake pads and rotors to optimize their performance and ensure proper functionality. During burnishing, the brake pads and rotors undergo a series of controlled braking cycles to transfer a thin layer of friction material onto the rotor surface. This helps in enhancing the contact and friction between the brake pads and rotors, improving braking efficiency and reducing the risk of brake noise and vibration. Burnishing ensures proper conditioning of brake pads and rotors to optimize performance and establish a consistent frictional characteristics between brake components. It also enhance the durability and longevity of the braking system components for reliable performance over time.

**Table 4:** Burnishing test results of sintered brake pad.

Burnishing cycles @ 65 kmph (Sintered)					
Cycle no.	Deceleration (g)	Pressure (Bar)	Torque (Kg-m)	Stopping distance (m)	Stopping time (sec)
1	0.37	24.77	20.52	48.14	5.42
5	0.36	24.66	20.48	48.36	5.42
10	0.37	24.28	20.54	48.02	5.42
15	0.37	24.74	20.57	48.16	5.42
20	0.36	24.65	20.48	48.20	5.42
25	0.37	24.22	20.56	48.08	5.42
30	0.37	24.77	20.52	48.14	5.38
35	0.36	24.65	20.47	48.36	5.42
40	0.37	24.37	20.52	48.09	5.42
45	0.37	24.81	20.53	48.13	5.44
50	0.36	24.65	20.47	48.34	5.42

The burnishing test results of sintered brake pad in Table 4 and organic brake pad in Table 5. reveal differences in performance between sintered and organic brake pads. Sintered brake pads show higher torque, pressure resistance, and deceleration, indicating superior durability and braking efficiency. They also exhibit lower stopping time and distance, making them more effective in high-performance applications. Organic brake pads, however, display higher stopping distances and lower torque, suggesting faster wear but smoother braking at lower speeds.

**Table 5:** Burnishing test results of organic brake pad.

Burnishing cycles @ 65 kmph (Organic)					
Cycle no.	Deceleration (g)	Pressure (Bar)	Torque (Kg-m)	Stopping distance (m)	Stopping time (sec)
1	0.37	27.03	20.77	48.65	5.53
5	0.37	27.05	20.82	48.52	5.51
10	0.37	28.40	20.34	48.89	5.51
15	0.37	28.37	20.68	48.32	5.52
20	0.37	29.58	20.26	48.65	5.53
25	0.37	29.27	20.42	48.29	5.51
30	0.37	27.38	20.94	48.47	5.43
35	0.37	27.23	20.51	48.94	5.45
40	0.37	26.46	20.45	48.72	5.45
45	0.37	26.62	20.25	48.54	5.45
50	0.37	26.89	20.29	48.14	5.44

While organic pads are quieter and provide better initial bite, sintered pads are more reliable for extreme conditions. The results highlight that sintered pads are better suited for demanding environments, such as racing or heavy-duty applications.

### Lever load test

The test aims to establish a correlation between load on the primary brake lever, lever travel, and hydraulic pressure generated for brake actuation, ranging from roughly 30 N to 280 N. Instead of generating hydraulic pressure internally, the dynamometer uses a complete disk brake assembly, ensuring real-world conditions. Prior to the experiment, brake bleeding eliminates air pockets for accurate results. An additional fixture with an electronically operated piston applies predetermined loads to the brake lever. Pressure gauges on the brake hose measure pressure, while an actuator tracks lever travel, ensuring adherence to acceptance criteria where pressure drop shouldn’t exceed 20% of the theoretically calculated pressure.

**Fig. 17:**
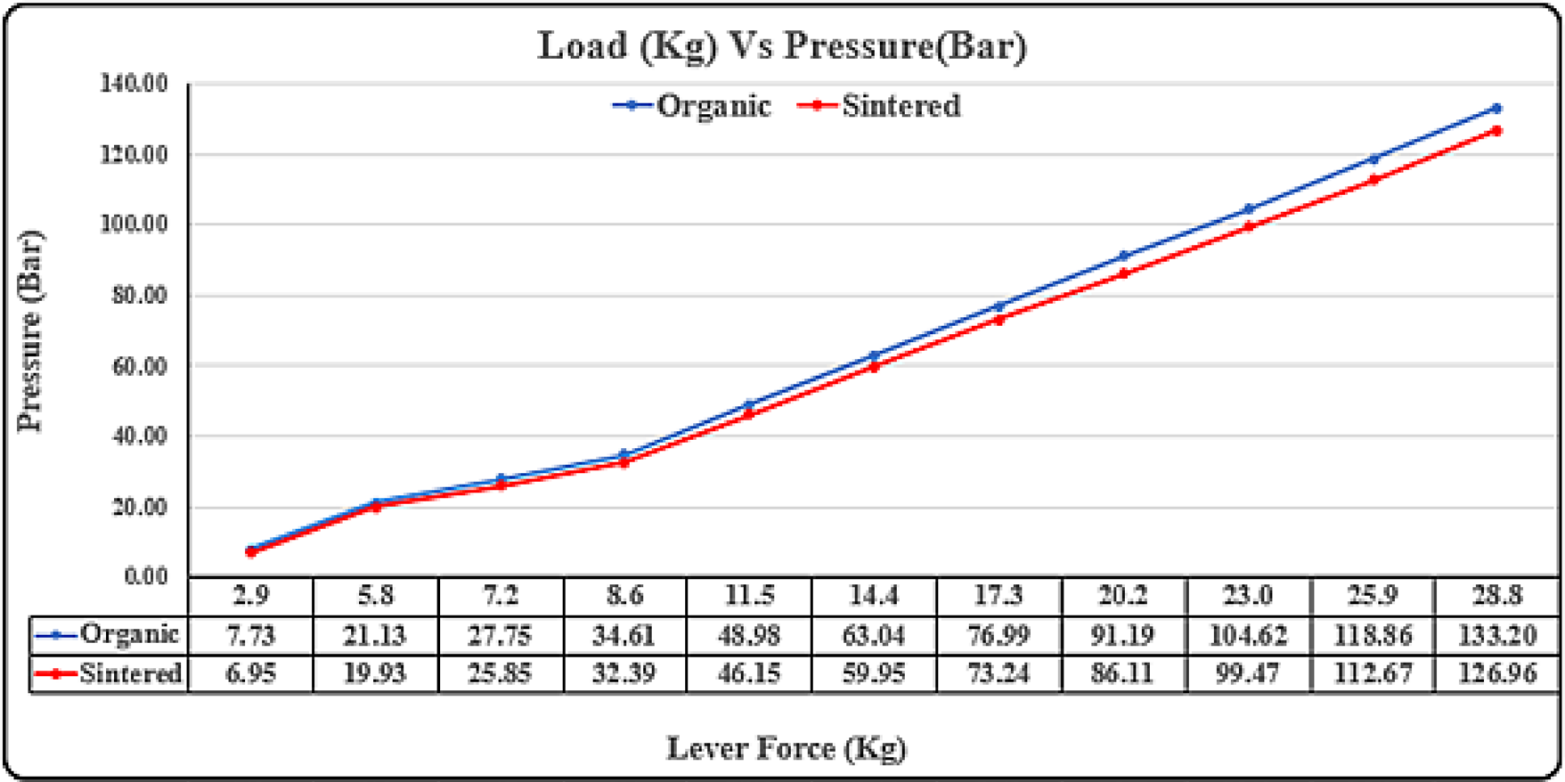
Lever load vs pressure test.

The load vs. pressure graph in Fig. 17. indicates that sintered brake pads endure less pressure for the same load, extending component lifespan and improving driver safety during panic braking. Additionally, sintered pads show less travel, crucial for overall brake system longevity and durability.

**Fig. 18:**
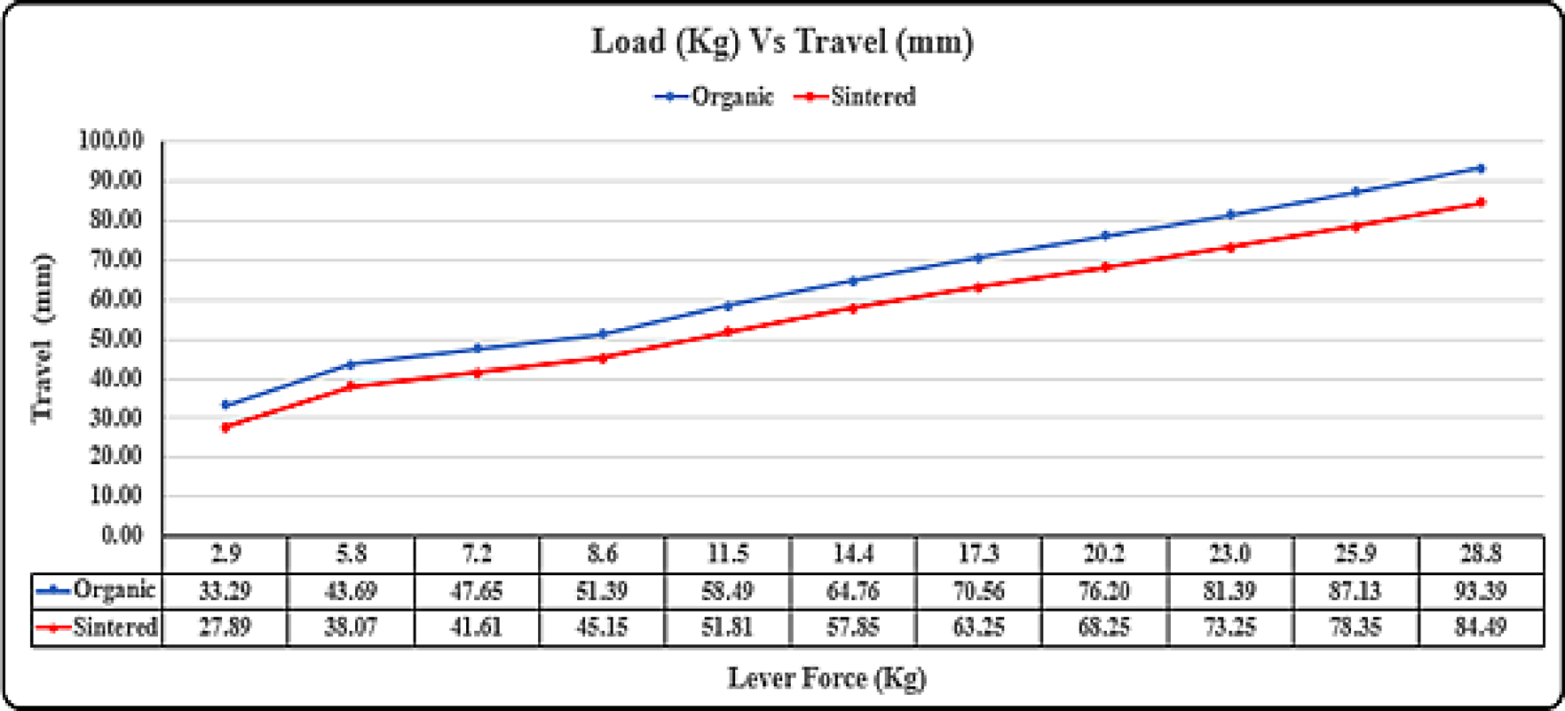
Lever load vs travel.

The load vs. travel graph in Fig. 18 indicate that the sintered brake pads endure less travel for the same load, allowing driver to exert less force and improving overall brake system longevity and durability. Sintered brake pads exhibit lower pressure in the caliper assembly for the same load, reducing fatigue on parts and extending their lifespan. During high-load situations like panic braking, sintered pads require significantly less force, enhancing driver safety by facilitating emergency braking with ease. Sintered pads demonstrate less travel of the brake lever, indicating their efficiency in overall braking action, essential for vehicle safety and durability of the brake caliper system. While marginal differences may exist at lower forces, the substantial benefits of sintered pads become pronounced in critical braking scenarios, underlining their superiority in enhancing both safety and longevity of brake systems.

### Friction coefficient test

Friction material testing is designed to evaluate the performance of friction materials used in brakes, clutches, and other applications by subjecting them to various test conditions. It assesses the wear characteristics and durability of friction materials under simulated operating conditions. The machine can perform dynamic tests to simulate the frictional forces experienced during braking or clutch engagement. It collects data on parameters such as friction coefficient, wear rate, temperature, and surface characteristics to analyze the performance of friction materials.

**Fig. 19:**
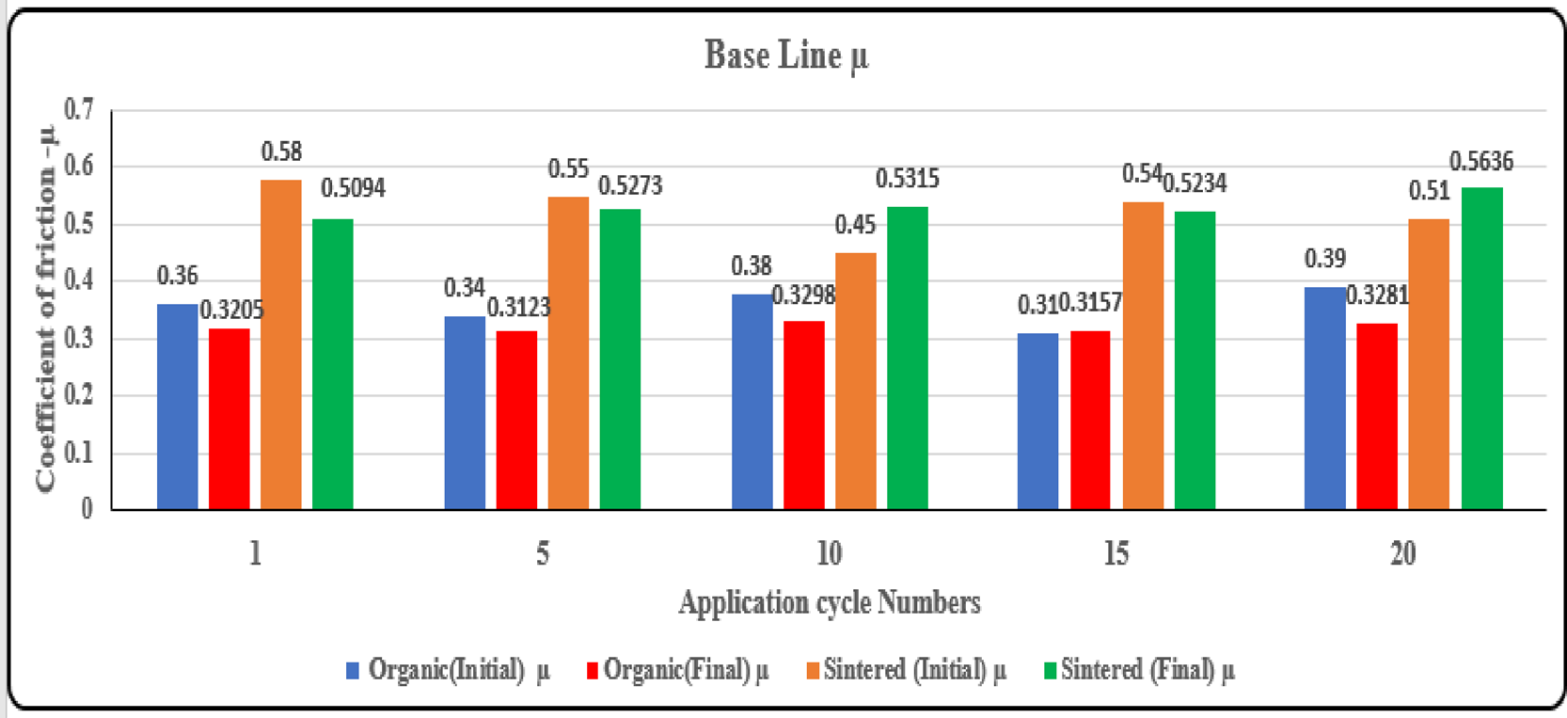
Coefficient of friction variation for sintered and organic brake pads across test cycles.

It can be inferred from the Fig. 19 that the sintered brake pads exhibit a higher coefficient of friction compared to organic pads in both initial and final conditions, indicating superior braking performance and stability over multiple cycles. Organic pads show a noticeable increase in friction over cycles, suggesting surface conditioning and material adaptation with use. Sintered pads demonstrate consistent frictional behavior, with final µ values remaining relatively stable, highlighting their durability. Additionally, the final µ values are higher than the initial ones for both materials, signifying improved braking performance as the pads undergo wear and surface optimization.

### Material composition

The performance disparity between organic and sintered brake pads can be directly linked to their intrinsic material compositions and the associated tribological mechanisms. Organic brake pads typically employ a phenolic resin matrix reinforced with organic fibers (e.g., aramid/Kevlar) and mineral fillers such as barite or vermiculite. These constituents impart good vibration damping and noise suppression, making them favorable for comfort-oriented applications. However, the organic matrix has limited thermal stability, softening at elevated temperatures and accelerating wear. The lower thermal conductivity of these materials also traps heat at the friction interface, promoting fade during prolonged or high-energy braking events.

In contrast, sintered brake pads are manufactured from a blend of metallic powders commonly copper, iron, and occasionally bronze bonded under high temperature and pressure. The metallic matrix provides superior thermal conductivity, efficiently dissipating heat into the rotor and ambient environment, thereby minimizing thermal fade. Hard metallic particles improve load-bearing capacity, allowing sintered pads to maintain structural integrity under high contact pressures. The inclusion of graphite or other lubricating phases within the metallic matrix helps stabilize the coefficient of friction, reducing wear on both pad and rotor surfaces (Bellini et al.).

Moreover, the wear debris generated by sintered pads tends to be fine and less prone to adhere to the rotor surface, preventing uneven frictional contact. Conversely, the fibrous and resinous debris from organic pads may smear or glaze, altering the effective contact area and contributing to inconsistent braking. Thus, the compositional differences particularly in thermal conductivity, mechanical hardness, and frictional stability directly explain the observed superior high-temperature performance, wear resistance, and consistency of sintered pads compared to organic alternatives (Mahale et al.).

## Conclusion

The results of this study provide valuable insights into the comparative performance of sintered and organic brake pads, reinforcing the importance of material selection in braking efficiency and durability.

- Superior Performance of Sintered Pads: Sintered brake pads demonstrated higher durability, with a wear mass loss of only 0.82 g after 1000 cycles compared to 1.35 g for organic pads. They retained a consistent coefficient of friction (average µ ≈ 0.42), shorter stopping distances (18.1 m) and times (1.74 **s**), and lower variability, making them highly suitable for high-performance and heavy-duty applications such as sports motorcycles and high-speed vehicles.
- Organic Pads’ Environmental and Noise Benefits: Organic brake pads, despite faster wear and reduced effectiveness under high-temperature conditions, offered smoother engagement and lower noise. However, their stopping distances averaged 19.7 m with times of 2.84 s, and they required up to 15% higher hydraulic pressure than sintered pads, which can increase caliper wear over time.
- Enhanced Heat Dissipation in Sintered Pads: The thermal stability of sintered brake pads was significantly higher than that of organic pads. They exhibited better heat dissipation characteristics, preventing brake fade and ensuring consistent performance over extended usage. Sintered pads operated at up to 10 °C cooler than organic pads at 0.1–0.3 g deceleration, reducing fade risk. Their metallic matrix improved heat conduction and structural stability under extended high-load braking.

Future implications and sustainable development: This study highlights the need for continued innovation in brake pad materials, focusing on sustainability, recyclability, and performance optimization. Future research should explore hybrid materials that combine the advantages of sintered and organic pads to develop eco-friendly, high-performance braking solutions. Additionally, advancements in biodegradable friction materials and energy-efficient manufacturing processes will be crucial in minimizing the environmental impact of brake pad production and usage.

## Data Availability

All data generated or analysed during this study are included in this published article.
